# Mechanism of Anomalous Anisotropic Colossal Magnetoresistance in Quasi‐2D Mn_3_Si_2_Te_6_ Bulk Single Crystal

**DOI:** 10.1002/advs.202514651

**Published:** 2025-10-14

**Authors:** Shiqi Li, Xiong He, Shuai Li, Tianyi Li, Wenhao Zhang, Lizhi Yi, Guangduo Lu, Zhengcai Xia, Yunli Xu, John Q Xiao, Liqing Pan

**Affiliations:** ^1^ Hubei Engineering Research Center of Weak Magnetic‐field Detection College of Science China Three Gorges University Yichang 443002 China; ^2^ Wuhan National High Magnetic Field Center Huazhong University of Science and Technology Wuhan 430074 China; ^3^ Department of Physics and Astronomy University of Delaware Newark DE 19716 USA

**Keywords:** anisotropic magnetoresistance, anomalous magnetotransport, colossal magnetoresistance, quasi‐2D magnetic material, spin‐dependent scattering

## Abstract

Mn_3_Si_2_Te_6_, quasi‐2D ferrimagnetic semiconductor, exhibits anomalous saturated colossal magnetoresistance (CMR) only when a magnetic field is applied along its magnetic hard magnetization axis, suggesting unconventional underlying physics and promising potential for spintronic applications. However, the intrinsic mechanism behind this anomalous anisotropic CMR remain unresolved. In this work, the temperature and angular dependencies of magnetoresistance (MR) in high‐quality Mn_3_Si_2_Te_6_ single crystals are systematically investigated. The MR measured within the easy *ab*‐plane shows no saturation, whereas a large negative saturation MR of ≈ −100% is observed along the hard magnetization *c*‐axis below the Curie temperature. To explain this behavior, a novel model is proposed in which in‐plane magnetic fields induce quasi‐2D magnetotransport, while out‐of‐plane fields promote a transition to 3D transport. Notably, when the *c*‐axis field exceeds the demagnetizing field, the alignment between spin‐polarized carriers and magnetic moments significantly suppresses scattering. The results challenge the applicability of the chiral orbital currents (COC) model in Mn_3_Si_2_Te_6_ single crystals and establish a new framework for controlling the CMR effect in layered magnets, offering a pathway toward future spintronic technologies.

## Graphical abstract

The external field exceeds H_d_, the speed polarization abruptly reorients to align with the Mn magnetic moments, leading to an MR ≈ −100%.

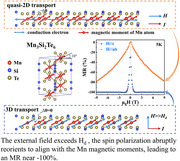

## 1 Introduction

Colossal magnetoresistance (CMR) is a phenomenon observed in certain strongly correlated electron materials, where an applied magnetic field induces a dramatic change in electrical resistance. Since its discovery in perovskite manganites in the 1990s,^[^ ^1^ ^]^ CMR has driven extensive research efforts in condensed matter physics and materials science. Materials exhibiting CMR mainly consist of perovskite manganites,^[^ ^2^, ^3^, ^4^, ^5^, ^6^, ^7^, ^8^, ^9^ ^]^ bilayer manganites,^[^ ^10^ ^]^ oxide spinels,^[^ ^11^ ^]^ phosphides,^[^ ^12^ ^]^ and certain sulfides.^[^ ^13^, ^14^ ^]^ The CMR effect in these materials is governed by strong electronic correlations, with key mechanisms including double‐exchange interactions, Jahn‐Teller distortions, and charge/orbital ordering.^[^ ^2^, ^4^, ^7^, ^8^, ^9^, ^15^, ^16^, ^17^, ^18^ ^]^ Recently, abnormal CMR has been observed in Mn_3_Si_2_Te_6_. Different from conventional CMR materials, the Mn_3_Si_2_Te_6_ exhibits a distinct behavior: the CMR effect that is prone to saturation only occurs when a magnetic field is applied along the magnetic hard axis, and is completely unsaturated when the field is aligned with the easy axis‐even under full magnetization saturation.^[^ ^19^, ^20^, ^21^ ^]^ This unusual CMR behavior suggests underlying unconventional physics and highlights the materials’ promise for future spintronic applications.

Mn_3_Si_2_Te_6_ is a quasi‐2D ferrimagnetic semiconductor (trigonal, space group $P\bar{3}1c$ (No. 163), *T*_C_≈78 K) with two inequivalent Mn sites (Mn1 and Mn2) forming a trimer‐honeycomb lattice. It exhibits canted ferrimagnetic order, with average magnetic moments of 4.55 μ_B_ for Mn_1_ and 4.20  μ_B_ for Mn_2_.^[^ ^22^ ^]^ Recent neutron diffraction results reveal a noncollinear magnetic structure below *T*_C_, where Mn spins are primarily confined to the *ab*‐plane but exhibit in‐plane canting of 10° toward the *c*‐axis under ambient conditions.^[^ ^22^ ^]^ Several characteristic properties of Mn_3_Si_2_Te_6_ have been identified to date, including anomalous CMR,^[^ ^19^, ^20^, ^21^ ^]^ anisotropic magnetoresistance (AMR),^[^ ^19^, ^23^ ^]^ the Nernst effect,^[^ ^20^ ^]^ a pressure‐induced metal‐to‐insulator transition,^[^ ^24^ ^]^ and the anomalous Hall effect (AHE).^[^ ^25^, ^26^ ^]^ Among these, the anomalous CMR effect stands out as particularly striking. Junho Seo et al.^[^ ^27^ ^]^ attribute this behavior to a spin orientation‐dependent topological band degeneracy. However, similar to conventional CMR models, this mechanism presumes spin polarization along the *c*‐axis. This raise an intriguing question: how can a small *c*‐axis magnetization component gives rise to significant scattering and, consequently, produce such a pronounced CMR effect. Recently, to explain the abnormal CMR effect, the chiral orbital currents (COC)‐induced orbital magnetic moments (***M_COC_***) model had also been proposed. Their interpretation suggests that the noncollinear magnetic structure of Mn_3_Si_2_Te_6_ is crucial for the formation of COC below *T*_C_. These COCs circulate along the edges of MnTe_6_ octahedra, primarily within the *ab*‐plane, giving rise to a net magnetic moment (***M*** *_COC_*) of ≈ 0.1 μ_B_ predominantly oriented along the *c*‐axis. When a magnetic field is applied along the *c*‐axis, the alignment of ***M*** *_COC_* also aligns the COCs, reducing scattering significantly and producing the anomalous CMR effect.^[^ ^21^ ^]^ The concept of COCs originates from early studies of superconductors, where persistent currents form due to the Meissner effect to expel external magnetic fields. COCs have been investigated in cuprates,^[^ ^28^, ^29^, ^30^, ^31^, ^32^, ^33^, ^34^ ^]^ iridates,^[^ ^35^, ^36^, ^37^ ^]^ and kagome superconductors.^[^ ^38^, ^39^, ^40^ ^]^ However, in conventional magnetic material systems, identifying comparable mechanism that can generate such circulating currents remains challenging. Therefore, whether the COC model applies to Mn_3_Si_2_Te_6_, and whether it fully accounts for the observed anomalous anisotropic CMR, remains an open and compelling question.

In this work, high‐quality Mn_3_Si_2_Te_6_ single crystals were successfully synthesized, and their magnetic properties, magnetoresistance (MR), and temperature‐dependent AMR were characterized systematically. The MR measured with the magnetic field in the easy magnetization *ab*‐plane shows little sign of saturation. While a strong negative MR, reaching ≈ 100%, is observed along the hard magnetization *c*‐axis, below the Curie temperature. To understand the origin of this anomalous anisotropic CMR effect, we analyze it in the context of the material's crystal and magnetic structure, offering an alternative perspective to the COC model. Additionally, AMR measurements reveal that the magnetization of Mn_3_Si_2_Te_6_ possesses a finite component along the *c*‐axis.

## 2 Results and Discussion

**Figure 1:**
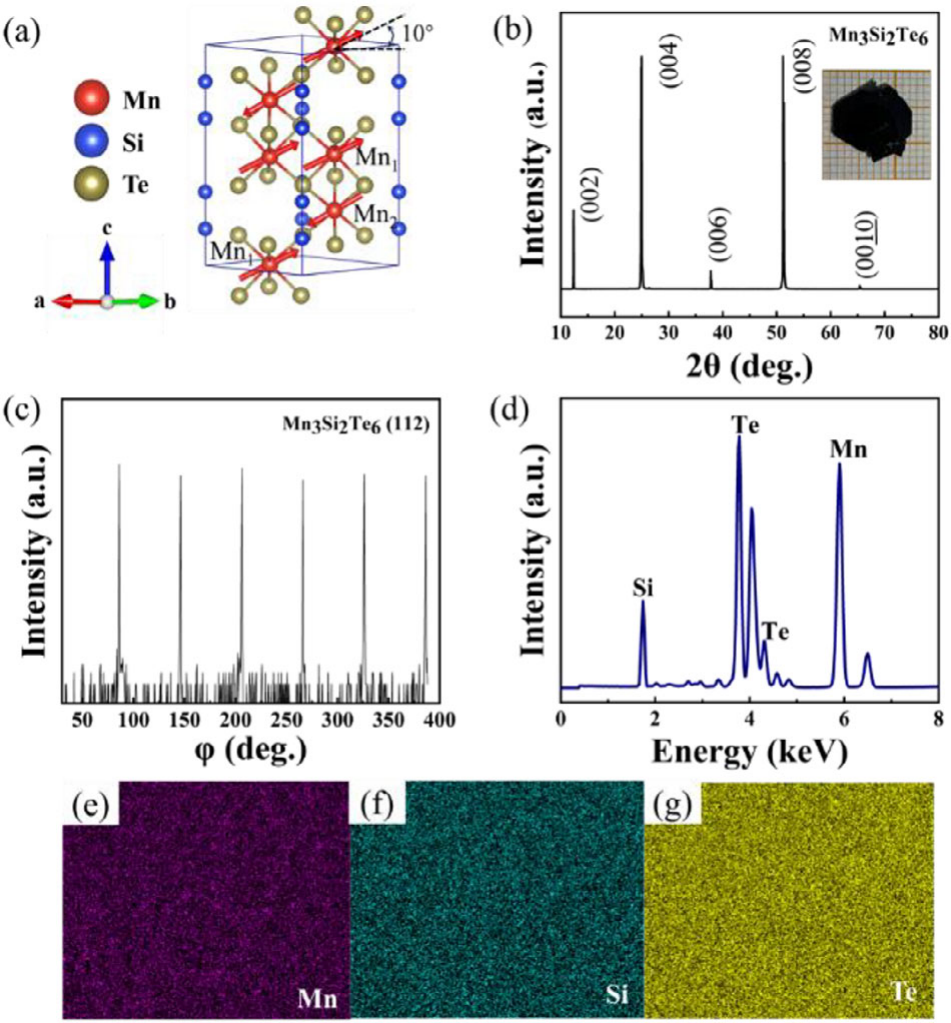
The structure diagram and characterization analysis results of the Mn_3_Si_2_Te_6_ single crystal. a) Crystal and magnetic structure of the synthesized Mn_3_Si_2_Te_6_. b) Room‐temperature XRD spectrum of the Mn_3_Si_2_Te_6_ single crystal. The inset is the sample photo. c) φ‐scan profile demonstrating the crystal's sixfold rotational symmetry. d) EDS spectrum of the Mn_3_Si_2_Te_6_. e–g) are the elemental distribution maps.

The crystal structure of Mn_3_Si_2_Te_6_ is illustrated in **Figure** **1**a. Notably, this compound exhibits a distinctive magnetic configuration characterized by ferromagnetic (FM) order within the *ab*‐plane and antiferromagnetic coupling along the *c*‐axis.^[^ ^41^ ^]^ Mn_3_Si_2_Te_6_ crystallizes in a layered trigonal structure featuring two inequivalent manganese sites, Mn_1_ and Mn_2_. The Mn_1_ ions form edge‐sharing MnTe_6_ octahedra, that create a honeycomb lattice within the *ab*‐plane, whereas the Mn_2_ ions occupy MnTe_6_ octahedra arranged in a triangular lattice interleaved between the honeycomb layers. This geometry produces a magnetically frustrated Mn network with three primary antiferromagnetic exchange interactions. The nearest‐neighbor interaction (**J_1_**) couples between Mn_1_ (honeycomb layer) and Mn_2_ (triangular layer) with a bond distance of 3.541(1) Å. The next‐nearest‐neighbor interaction (**J_2_**) takes place between Mn ions within the basal plane, separated by 4.056(1) Å. A third interaction (**J_3_**) connects Mn ions across adjacent layers at 5.401(1) Å. No structural transition or noticeable anomaly in the lattice constants is observed across the Curie temperature. Both Mn sites exhibit canting of ≈10°toward the *c*‐axis.^[^ ^22^ ^]^ The XRD diffraction patterns are shown in Figure 1b, indicating excellent [001] crystallographic orientation of the synthesized crystals. The crystal quality is further verified with in‐plane φ‐scan (Figure 1c), which reveals sixfold symmetry, as expected from trigonal systems. Chemical composition and spatial homogeneity were verified by elemental analysis. Quantitative EDS results (Figure 1d) yield an atomic ratio of Mn: Si: Te ≈ 3.0: 1.9: 6.1, consistent with the ideal stoichiometric ratio of 3:2:6 for Mn_3_Si_2_Te_6_. Moreover, elemental mapping (Figure 1e–g) demonstrates homogeneous spatial distribution of the Mn, Si, and Te elements throughout the crystal lattice.

**Figure 2:**
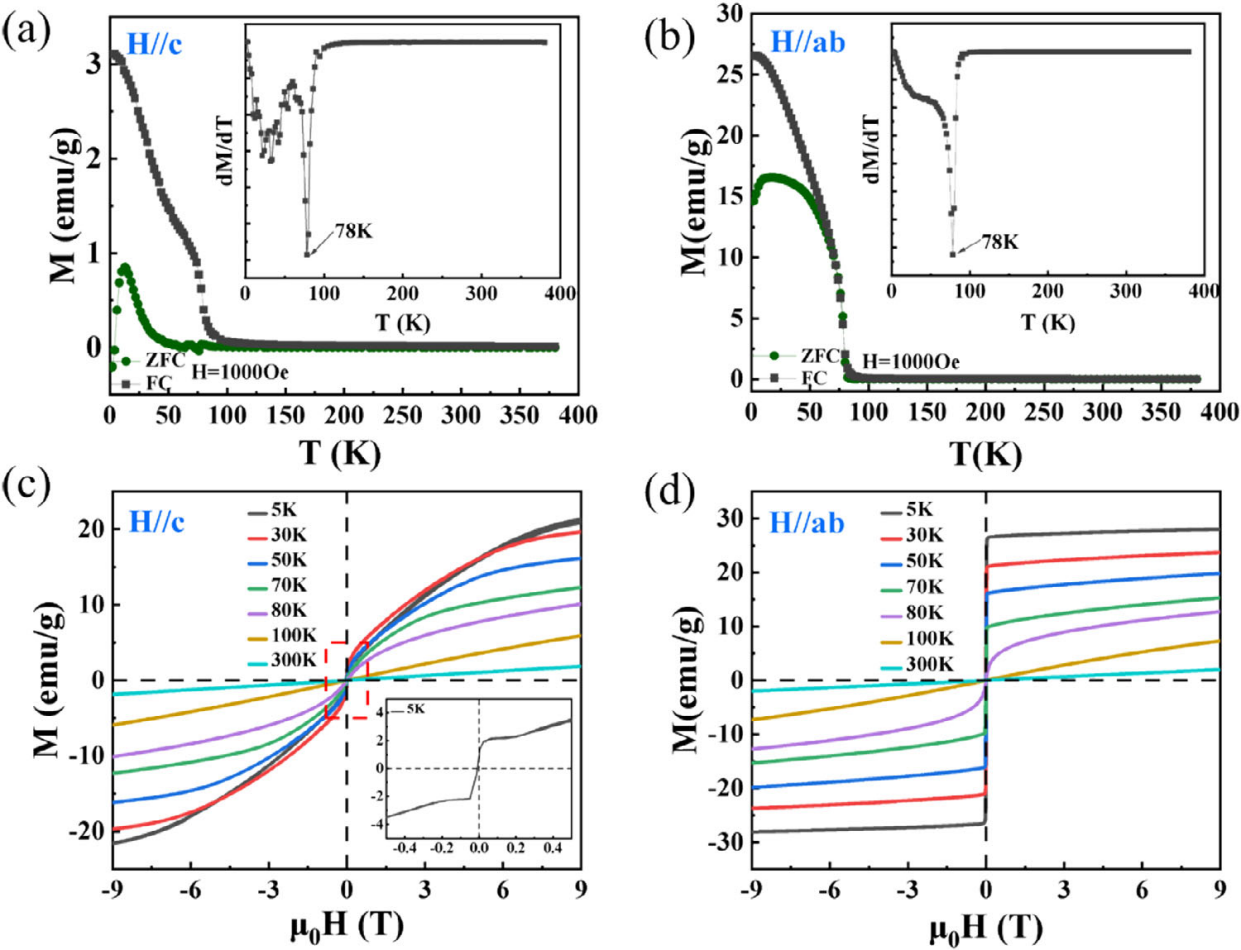
Magnetic properties of the Mn_3_Si_2_Te_6_ single crystal. a,b) The temperature‐dependent magnetization under zero‐field cooling (ZFC) and field cooling (FC) conditions along the *H*//*c*‐axis and the *H*//*ab*‐plane, with the field cooling magnetic field being 1000 Oe. Insets: Corresponding derivatives d*M*/d*T*. c,d) Magnetic hysteresis (*M*‐*H*) loops measured at indicated temperatures for (c) *H*//*c*‐axis and (d) *H*//*ab*‐plane. The inset in Figure 2c shows an enlarged image at a temperature of 5 K.

Systematic magnetic characterization of Mn_3_Si_2_Te_6_ single crystals reveals distinct anisotropic behavior. **Figure** **2**a,b presents the temperature‐dependent magnetization measured under zero‐field‐cooled (ZFC) and field‐cooled (FC) protocols with applied magnetic fields parallel to the *c*‐axis (*H*//*c‐*axis) and *ab*‐plane (*H*//*ab*‐plane), respectively. For *H*//*c‐*axis, a pronounced bifurcation between the ZFC and FC curves emerges at 78 K (Figure 2a), signifying the Curie temperature *T*_C_ (78 K). This *T*_C_ value is consistent with previous literatures.^[^ ^20^, ^41^, ^42^, ^43^ ^]^ In contrast, for *H*//*ab*‐plane (Figure 2b), the ZFC and FC curves remain superimposed over a broad temperature range due to the *ab*‐plane being the easy magnetization direction, where magnetic moments readily align under external fields. From the Curie‐Weiss analysis results on the selected FC data (see Figure S2, Supporting Information), the effective magnetic moment of Mn is 4.21 μ_B_ when *H* is parallel to the *ab*‐plane, and 3.74 μ_B_ when *H* is parallel to the *c*‐axis, respectively. Both values are slightly lower than the neutron diffraction characterization values of 4.2 and 4.5 μ_B_.^[^ ^22^ ^]^ Figure 2c,d displays the magnetic hysteresis loops measured at various temperatures with applied fields parallel to the *c*‐axis and *ab*‐plane, respectively. For *H*//*c*‐axis (Figure 2c), magnetization is not saturated even at the maximum applied field of 9 T. This lack of saturation reveals the strong interlayer antiferromagnetic coupling (**J**_1_). This competitive interplay between the Zeeman energy and the **J**_1_ exchange results in a magnetization process characteristic of ferrimagnetic behavior.^[^ ^44^ ^]^ In contrast, for *H*//*ab*‐plane (Figure 2d), the material exhibits clear easy‐axis magnetization below *T*c. Saturation magnetization (***M*** s) increases monotonically with decreasing temperature, consistent with the progressive strengthening of magnetic ordering as thermal fluctuations are suppressed.^[^ ^44^ ^]^ Above 100 K, the system shows a fully linear paramagnetic response, confirming that Mn_3_Si_2_Te_6_ single crystals transition completely into the paramagnetic state.

**Figure 3:**
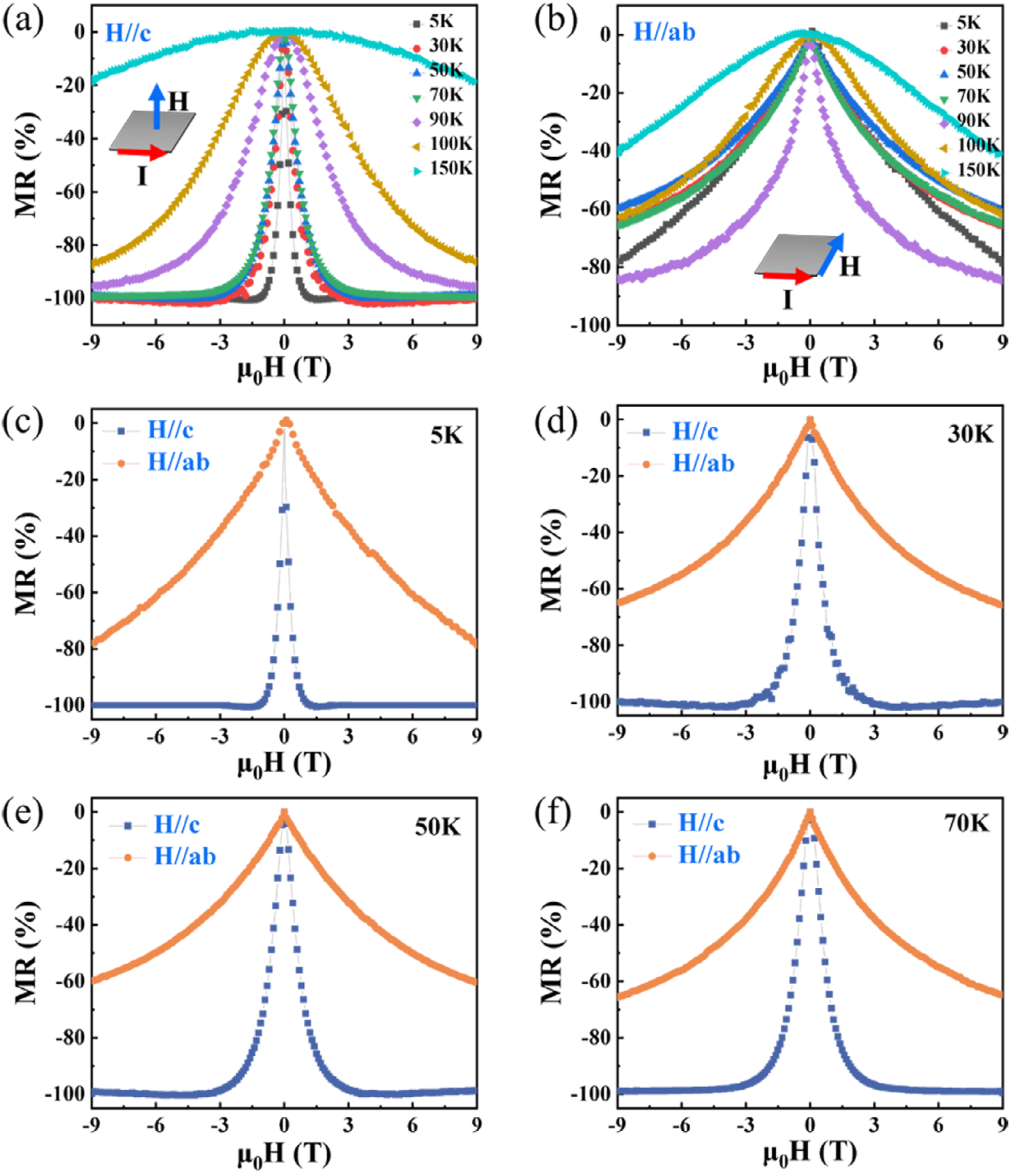
MR properties of the Mn_3_Si_2_Te_6_ single crystal. a,b) MR as a function of magnetic field magnitude under different field orientations. c–f) Comparison results of MR perpendicular and parallel to the *c*‐axis at different temperatures.

The MR properties of the Mn_3_Si_2_Te_6_ single crystal are shown in **Figure** **3**. In the case of *H*//*c*‐axis, when the temperature is 5 K, the resistance of the material changes from ≈ 3600 to 0.5 Ω, exhibiting a giant negative MR with strong temperature‐dependence (Figure 3a). The MR rapidly drops from 0% to ≈ −100% as the field increases from 0 to 9 T. In contrast, in the case of *H*//*ab*‐plane, when the temperature is 5 K, the material’ resistance varies from ≈ 1700 to 360 Ω. The MR in *ab*‐plane remains large and negative but does not saturate even at 9T, as shown in Figure 3b. The negative MR emerges via suppressing the spin fluctuations.^[^ ^45^ ^]^ The anisotropic MR behavior is further illustrated in Figure 3c–f, which compare the MR for *H*//*c*‐axis and *H*//*ab*‐plane at representative temperatures below the Curie temperature (5, 30, 50, and 70 K). Notably, the material exhibits an anomalous anisotropic MR: a large saturated negative MR along the magnetically hard magnetization *c*‐axis, in stark contrast to the non‐saturating MR along the easy *ab*‐plane. This behavior is opposite to what is typically observed in conventional CMR materials^[^ ^7^, ^8^, ^9^ ^]^ and is consistent with previous reports on Mn_3_Si_2_Te_6_.^[^ ^19^, ^20^, ^21^ ^]^

**Figure 4:**
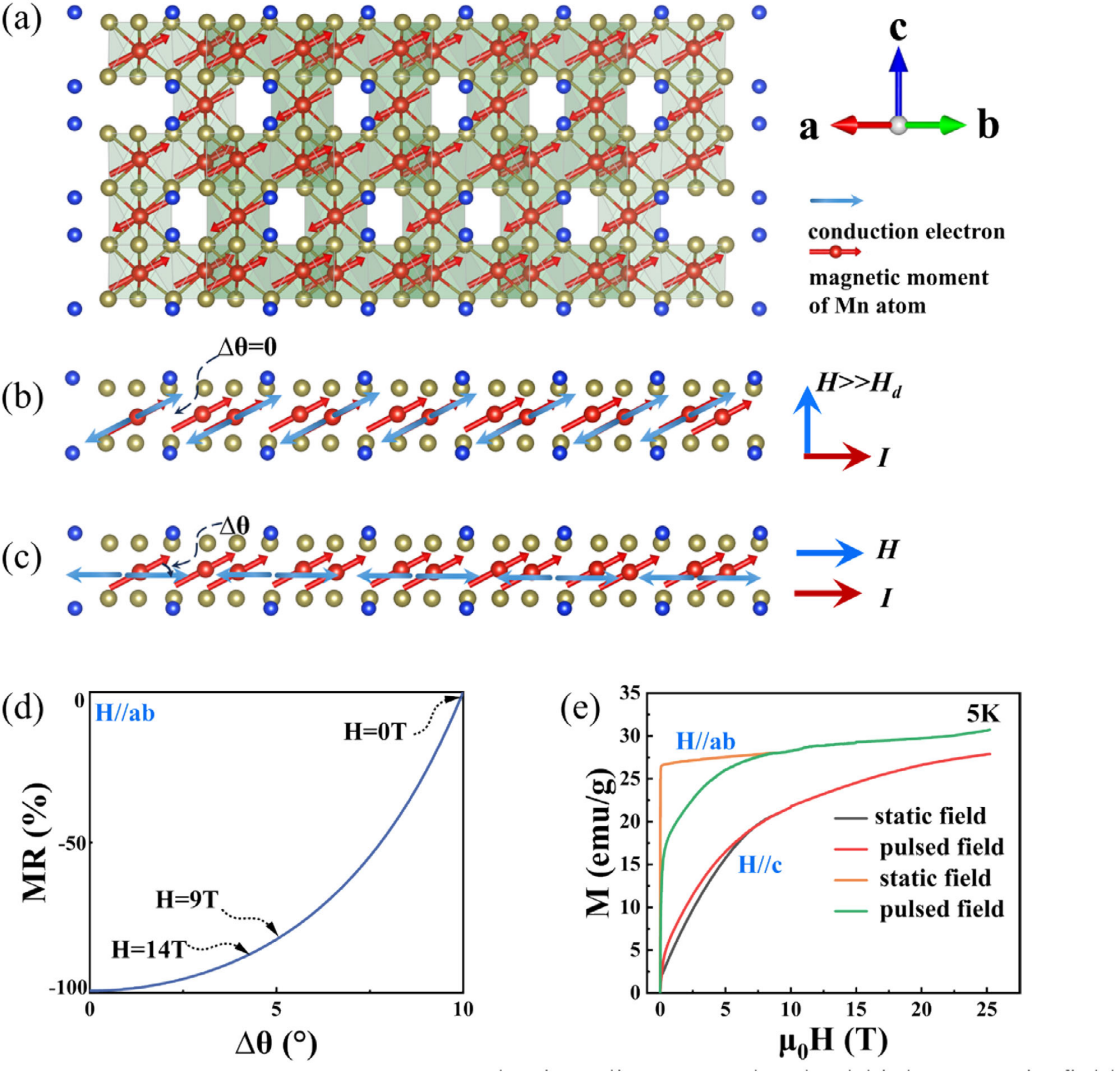
Anomalous anisotropic CMR mechanism diagram and pulsed high magnetic field data. a) Lattice and magnetic ordering of quasi‐2D Mn_3_Si_2_Te_6_ crystal. b) The situation where the magnetic field is applied along the *c*‐axis. c) The situation where the magnetic field is applied along the *ab*‐plane. [(a) and (b)] The red arrows on the Mn atoms represent the atomic magnetic moments, and the blue arrows represent the polarization direction of the conduction electrons. d) MR as a function of the angle between the direction of carrier spin polarization and Mn magnetic moment. e) High‐field magnetization data were collected with the magnetic field applied both parallel (*H*//*c*‐axis) and perpendicular (*H*//*ab*‐plane) to the crystallographic *c*‐axis.

To elucidate the physical origin of this anomalous anisotropic CMR, we systematically investigate whether the *c*‐axis magnetization component can induce sufficient carrier scattering to account for the observed giant CMR effect. As reported by Ye et al.,^[^ ^22^ ^]^ the magnetic moments in the Mn_3_Si_2_Te_6_ exhibit a ground‐state tilt of 10° from the *ab*‐plane in the absence of an external field. As shown in **Figure** **4**a, applying in‐plane magnetic fields does not fully align the Mn spins within the *ab*‐plane, leaving a residual tilt toward the *c*‐axis. This is further supported by the inset of Figure 2c, where a sharp initial increase in magnetization at low fields suggests a pre‐existing out‐of‐plane tilt. As a result, a finite *c*‐axis magnetization component persists (Figure 4a), which leads to strong anisotropic carrier scattering. This spin‐dependent scattering mechanism gives rise to the characteristic non‐saturating MR observed under *H*//*ab*‐plane. When the field is applied along the *c*‐axis, increasing field strength progressively tilts spins toward the *c*‐axis, though full saturation is not achieved. Regarding the lattice contribution: when a sufficiently strong external magnetic field, for example along the *c*‐axis, is applied, the spin polarization direction of the conduction electrons becomes aligned with the Mn atomic magnetic moments (Figure 4b). Under this condition, spin‐dependent scattering is strongly suppressed, and the residual resistance arises primarily from the lattice scattering. Our measurement results show that the lattice contribution is very small. Therefore, the spin‐dependent scattering is the dominant mechanism responsible for the CMR effect in the Mn_3_Si_2_Te_6_. To elucidate the mechanism underlying this anisotropic scattering and MR, we further analyze the system with the linear‐response theory framework.

The magneto‐conductivity can be found from the general Kubo Equation (1),^[^ ^46^ ^]^

(1) $$\sigma_{aa}=\frac{\pi \hbar e^{2}}{V}\sum_{u,u^{\prime }}\int \frac{\partial n\left(\omega \right)}{\partial \omega }A_{u}\left(\omega \right)A_{u^{\prime }}\left(\omega \right){\left|\langle u^{\prime }\left|v_{a}\left|u\right.\right.\rangle \right|}^{2}d\omega$$

where the subscript *aa* denotes that both the excitation current and the voltage measurement are along *a*‐direction. The effect of the magnetic field is incorporated in the quantum state |*u*〉 and |*u*′〉, as well as the spectral functions *A_u_* (ω) and $A_{u^{\prime }}\left(\omega \right)$. Here, *V* denotes the volume of the system; *n*(ω) is the Fermi‐Dirac distribution function, and *v_a_* is the velocity operator along *a* direction. Given that the Hall signal in Mn_3_Si_2_Te_6_ is much smaller than the MR,^[^ ^21^, ^26^ ^]^ the magneto‐conductivity σ_*aa*_ is the decisive factor determining the MR.

In Equation (1), the integral over the energy ω and the partial derivative of the Fermi‐Dirac distribution function imply the conductivity is mainly contributed by carriers near the Fermi energy when the temperature is not very high. Based on this and the electronic structure of ferrimagnetic Mn_3_Si_2_Te_6_ calculated by density functional theory,^[^ ^27^ ^]^ one can infer that, the states |*u*〉 (also |*u*′〉) are from spin‐polarized Te‐p valence band or Mn‐d conduction band.

The spectral function is defined as $A_{u}\left(\omega \right)=i\left[G_{u}^{R}\left(\omega \right)-G_{u}^{A}\left(\omega \right)\right]/2\pi$, and the retarded and the advanced Green functions are $G_{u}^{R}\left(\omega \right)={\left[\omega -H-\Sigma_{u}\right]}^{-1}$ and $G_{u}^{A}\left(\omega \right)={\left[G^{R}\left(\omega \right)\right]}^{†}$, respectively. Here, H is the system Hamiltonian. Σ_*u*_ is the self‐energy of state |*u*〉, through which the scattering effect is considered. The scattering rate is proportional to the imaginary part of the self‐energy.^[^ ^47^ ^]^ Within the first Born approximation, the self‐energy is given by

(2) $$\begin{array}{c} \Sigma_{u}=\sum_{u^{\prime }}\frac{{\left|\langle u^{\prime }|M_{sp}|u\rangle \right|}^{2}}{\omega -E_{u^{\prime }}+i\eta } \end{array}$$

where *M_sp_* denotes the general scattering potential, *E*_*u*′_ is the energy of state |*u*′〉, and η is an infinitesimal positive constant. Since the spin‐dependent scattering dominates in our system, we focus on the spin components in the above formulas. One has

(3) $$\begin{array}{c} |u\rangle \propto \left(\begin{array}{c} \cos\frac{\theta }{2}\\ e^{i\phi }\sin\frac{\theta }{2} \end{array}\right) \end{array}$$

(4) $$\begin{array}{c} M_{sp}\propto \sin\theta^{\prime }\cos\phi^{\prime }\sigma_{x}+\sin\theta^{\prime }\sin\phi^{\prime }\sigma_{y}+\cos\theta^{\prime }\sigma_{z} \end{array}$$

where θ and ϕ denote the spin polarization angles of the carriers, and θ′and ϕ′ correspond to the orientations of the local Mn magnetic moments in the Mn_3_Si_2_Te_6_.

Under an applied magnetic field, the carriers become uniformly spin polarized. From the scattering matrix in Equation (2), we can define an effective transition amplitude *T_t_*,

(5) $$\begin{array}{ccc} T_{t} & = & {\left|\left(\begin{array}{cc} \cos\frac{\theta }{2} & e^{-i\phi }\sin\frac{\theta }{2} \end{array}\right)\left(\begin{array}{cc} \cos\theta^{\prime } & e^{-i\phi^{\prime }}\sin\theta^{\prime }\\ e^{i\phi^{\prime }}\sin\theta^{\prime } & -\cos\theta^{\prime } \end{array}\right)\left(\begin{array}{c} \cos\frac{\theta }{2}\\ e^{i\phi }\sin\frac{\theta }{2} \end{array}\right)\right|}^{2}\\ & = & \begin{array}{c} {\left(\cos\theta \cos\theta^{\prime }+\cos\left(\phi -\phi^{\prime }\right)\sin\theta \sin\theta^{\prime }\right)}^{2} \end{array} \end{array}$$

This effective transition amplitude reaches its maximum value *T_t_* = 1 when the spin polarization of carriers aligns with the magnetic moment of Mn atoms, i.e., when θ = θ′ and ϕ = ϕ′.

In Mn_3_Si_2_Te_6_, carriers undergo multiple scattering events during the transport process. The *T*‐matrix approximation for self‐energy provides a more accurate descriptions of scattering by including higher‐order terms beyond the first Born approximation (proportional to |〈*u*′|*M_sp_* |*u*〉|^2*n*^ with *n* ≥ 2). This further brings an effective scatting times *n* for the transition amplitude *T_t_*. By relating magneto‐conductivity σ_*aa*_ to the spectral function, Green function and self‐energy, and noting that MR is approximately inversely proportional to σ_*aa*_, one has the relation

(6) $$\begin{array}{c} MR=F\left(\frac{1}{{T_{t}}^{n}}\right) \end{array}$$

where *F* is a function positively correlated with $\frac{1}{{T_{t}}^{n}}$, and *n* is the effective number of scattering events.

When a relatively low magnetic field is applied in the *ab*‐plane at 5 K, the magnetization does not reach saturation immediately. A weak field merely rotates the magnetic moments, which initially point in various directions on the easy‐magnetization cone, toward positions on the same cone that are closer to the external field. In this regime the moments retain their original tilt of ≈ 10° relative to the *ab*‐plane. Thus, θ ≡ 0, due to the quasi‐2D nature of the system. Assuming that the scattering potential (Mn magnetic moments) tilts out of the plane by a small angle Δθ, i.e., θ′ = Δθ, and the azimuthal angles are aligned (ϕ = ϕ′), the MR becomes.

(7) $$\begin{array}{c} MR=F\left({\left(\frac{1}{\cos\left(\Delta \theta \right)}\right)}^{2n}\right)\propto F\left({\left(1+\frac{1}{2}{\left(\Delta \theta \right)}^{2}\right)}^{2n}\right) \end{array}$$

indicating that even small deviations of Mn moment orientation from the *ab*‐plane can cause appreciable changes in MR.

To estimate Δ*θ* under various field strengths, we model the magnetization response by considering the torque $\vec{L}=\vec{M}\times \vec{B}$ and the work *W* = *L* · δ acting on the magnetic momen. For a system where the exchange interaction between spins s_1_ and s_2_ is *J*, the work done by external field is *W* = −2*J* · *S* ^2^ *cos*δ, where *δ* is the rotation angle of the Mn moment. Balancing these contributions gives $\delta =\frac{BMsin\varphi }{JS^{2}}$ where φ is the angle between $\vec{B}$ and $\vec{M}$. Based on the experimental *M*‐*H* results and the theoretical saturation magnetization, we obtain $\frac{d\delta }{dB}=6.357^{\circ }/T$. At *μ*_0_ *H* = 9 T, this gives *δ* = 5.0°, corresponding to a tilt of 5.0° out of the *ab*‐plane; at 14 T, δ = 6.1°, with a tilt of 3.9°. Assuming $\rho_{\Delta \theta =0^{\circ }}\ll \rho_{\Delta \theta =10^{\circ }}$ and taking ρ(9T) ≈ 20%ρ(0T), as observed in Figure 4c, we estimate the effective scattering number *n*≈31. Using Eqs. (5) and (6), the dependence of MR on the angle between the carrier spin and the Mn moment is plotted in Figure 4d.

As the field strength increases, the magnetic moments gradually overcome the spin‐orbit exchange coupling and begin to align with the external field. Because symmetry breaking in this quasi‐2D Mn_3_Si_2_Te_6_ magnet confines the spin polarization of the conduction electrons strictly to the *ab*‐plane (Figure 4c), there remains a persistent ∼5° misalignment between the conduction‐electron spins and the magnetic moments even at 9 T. This slight misalignment, after multiple scattering events, gives rise to the observed CMR effect.

To further investigate the physical mechanisms underlying the observed MR behavior, we analyze the crystal and magnetic structure of Mn_3_Si_2_Te_6_. Given its quasi‐2D nature, we model each Mn‐containing layer as an infinite 2D slab, a valid approximation for estimating the demagnetizing field. The demagnetizing field is given by: $H_{d}=N\frac{I}{\mu_{0}}$, where the demagnetization factor *N* = 1 for an ideal thin plate perpendicular to the applied field. Using the per‐Mn magnetic moment 4.5μ_*B*_, as determined by neutron diffraction,^[^ ^22^ ^]^ we estimate the maximum demagnetizing field *H*_d_ for a single Mn atom to be as high as 1.6 T. When the magnetic moment is titled 10° from the *ab*‐plane, the remanent magnetic field is 0.28 T, placing the effective retreating field range between 0.28 and 1.6 T.

For out‐of‐plane (*H*//*c*‐axis) magnetic fields below this demagnetizing field, the spin polarization remains predominantly in the *ab*‐plane. However, once the external field substantially exceeds the demagnetization field of the quasi‐2D Mn_3_Si_2_Te_6_, the electron spins reorient toward the direction of the Mn atomic magnetic moments, no longer constrained by the strong in‐plane spin limitation characteristic of quasi‐2D transport (Figure 4b), maximizing the transition amplitude *T_t_* = 1. Consequently, the resistivity ρ_*H*_ *at* *H* ≫ *H_d_* becomes much lower than its zero‐field value, leading to an *MR* near − 100%. Magnetization measurements along the *c*‐axis show a continuous increase with applied field strength. According to the circulating orbital current (COC) model proposed by Zhang et al.,^[^ ^21^ ^]^ an additional magnetic contribution ***M*** *_COC_* could enhance the total magnetization. If this model were applicable to Mn_3_Si_2_Te_6_, the maximum magnetization would equal the theoretical saturation plus the COC contribution. Given that the reported circulating current per unit cell is 10, with each current contributing 0.1 μ_B_, the calculated maximum magnetization would reach ≈ 30 emu g^−1^. However, our high‐field data (Figure 4e) show that the measured values for *H*//c remain well below this theoretical limit. Additionally, the magnetization along *H*//c is significantly smaller than that observed along *H*//*ab* (Figure 4e). Very recently, no definitive evidence of the ***M*** *_COC_* is observed in Fang et al.’ study.^[^ ^48^ ^]^ Meanwhile, Joule heating effects may also contribute to the anomalous CMR effect.^[^ ^42^, ^48^ ^]^ Based on the above analysis, we believe that the COC model does not adequately describe Mn_3_Si_2_Te_6_ single crystals.

In addition, as shown in Figure S1 (Supporting Information), the trigonal crystal structure of Mn_3_Si_2_Te_6_ features non‐collinear Mn‐Te‐Mn bonds and a honeycomb arrangement of MnO_6_ octahedra‐distinct from the corner‐sharing MnO_6_ octahedra found on perovskite manganites. Consequently, the CMR mechanism differs from both the conventional double‐exchange and Jahn‐Teller distortion models.^[^ ^8^, ^9^ ^]^ Apart from the double‐exchange and Jahn‐Teller distortion, the electronic phase separation mechanism is most experimentally supported in the perovskite manganites system.^[^ ^7^ ^]^ In that scenario the antiferromagnetic charge‐ordered insulating phase competes with the ferromagnetic metallic phase; under an applied magnetic field the system transitions to the ferromagnetic metallic state, producing a characteristic inflection in the magnetization (*M*–*H*) curve.^[^ ^7^ ^]^ In our Mn_3_Si_2_Te_6_ crystals, no such transition is observed (Figure 2c,d). The *M*‐*H* curves show only a low‐field inflection that arises from the reorientation of the magnetic moment along different easy axes. Under a weak field, the moment initially aligns along the in‐plane directions closest to the field, and with increasing field they gradually rotates toward the field direction until saturation. This low‐field feature is not related to any magnetic phase transition, and the MR curves show no corresponding anomaly in this region. Even at higher fields no additional inflection points appear. Moreover, electronic phase separation in perovskite manganites requires charge ordering arising from mixed valence states such as Mn^+3^/Mn^+4^ or Fe^+2^/Fe^+3^.^[^ ^8^, ^9^ ^]^ In contrast, Mn ions in Mn_3_Si_2_Te_6_ retain a stable +2 valence,^[^ ^49^, ^50^ ^]^ which rules out the formation of charge‐ordered phase. Therefore, the CMR mechanism in Mn_3_Si_2_Te_6_ differs fundamentally from all three conventional models. Bases on our detailed theoretical analysis, we conclude that electron‐correlation‐driven spin‐dependent scattering is the dominant origin of the anomalous anisotropic CMR in this Mn_3_Si_2_Te_6_ materials.

**Figure 5:**
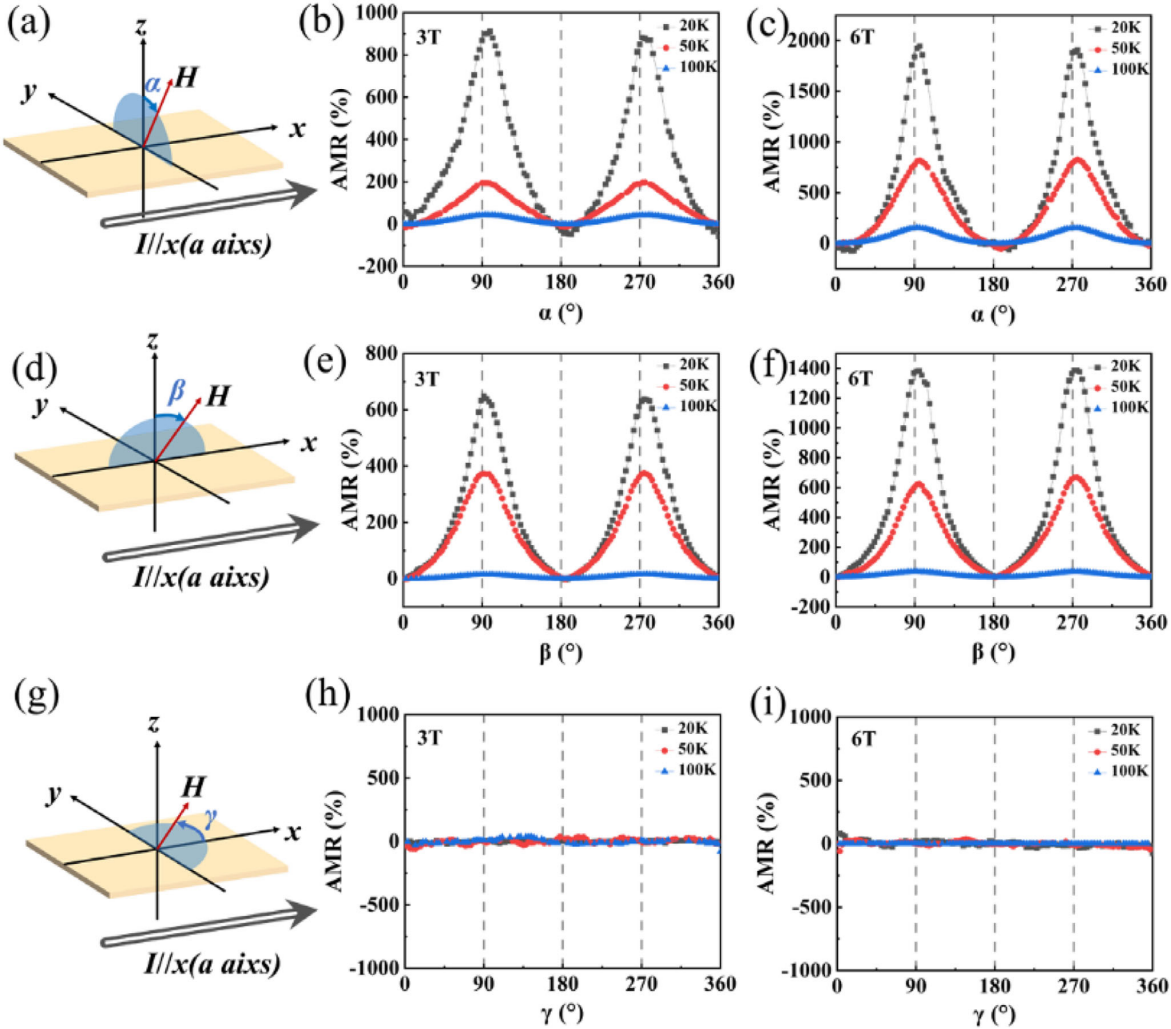
The AMR properties of the Mn_3_Si_2_Te_6_ single crystal. a) Schematic diagram illustrating the AMR measurement configuration in the *yz*‐plane. b,c) Angle‐dependent AMR data measured at magnetic fields of 3 T and 6 T, respectively. d) Schematic diagram illustrating the AMR measurement configuration in the *xz*‐plane. e,f) Angle‐dependent AMR results measured at 3 and 6 T, respectively. g) Schematic diagram illustrating the AMR measurement configuration in the *xy*‐plane. h,i) Angle‐dependent AMR data measured at magnetic fields of 3 and 6 T, respectively.

To further verify the presence of a finite magnetization component along the *c*‐axis, we performed angular‐dependent anisotropic magnetoresistance (AMR) measurements (**Figure** **5**). The magnetic field was applied in three principal planes (*yz*, *xz*, and *xy*), labeled as rotation angle *α*, *β*, and *γ*, respectively (Figure 5a,d,g). Herein, the *x*, *y*, and *z* axes correspond to the [010], [210], and [001] crystallographic directions, respectively. Figure 5b,c displays the angular dependence of the AMR measured in the *yz* plane under various constant magnetic fields, revealing clear twofold symmetry. The AMR amplitude gradually dimishes with increasing temperature. The angular dependence can be empirically described by:^[^ ^51^ ^]^

(8) $$\begin{array}{c} AMR=\left(\frac{R_{xx,\theta }-R_{xx,0}}{R_{xx,0}}\right)\times 100\% \end{array}$$

where *Rxx*,_θ_ is the longitudinal resistance at angle θ, and R*xx*,_0_ is the resistance at θ = 0.

Figure 5e,f depicts the angular dependence of the AMR within the *xz* plane, which shows weaker anisotropy compared to the *yz* plane, although a similar temperature dependence is observed. In contrast, AMR in the *xy* plane (Figure 5h,i) exhibits minimal variation, indicating that, MR within the *ab*‐plane is largely insensitive to the in‐plane orientation of the magnetic field relative to the current. This result reinforces the conclusion that the dominant spin orientation lies near the *ab*‐plane with a measurable tilt toward the *c*‐axis.

Although Mn_3_Si_2_Te_6_ has threefold crystallographic symmetry about the *c*‐axis, its easy magnetization direction lies on a conical surface tilted ≈80° from this axis. This configuration exhibits in‐plane isotropy (e.g., AMR*_xy_* ≈ 0) around the *c*‐axis and effectively breaks the original threefold rotation symmetry, giving rise instead to a fourfold symmetric AMR. The angular dependence of the MR is well‐described by the empirical formula, where ψ_*AMR*_ and φ_*AMR*_ denote the angles between the magnetization and the current direction, and between the magnetization and the [100] crystallographic axis, respectively:^[^ ^51^ ^]^

(9) $$\begin{array}{ccc} AMR & = & C_{I}\cos2\varphi_{AMR}+C_{2}\cos2\psi_{AMR}+C_{4}\cos4\psi_{AMR}\\ & & +\;C_{IC}\cos\left(4\psi_{AMR}-2\varphi_{AMR}\right) \end{array}$$

where the *C*_I_, *C*_2_, C_4_, and C_IC_ are phenomenological coefficients representing the noncrystalline term, twofold and fourfold crystalline terms, and a mixed crystalline/noncrystalline term, respectively.

**Figure 6:**
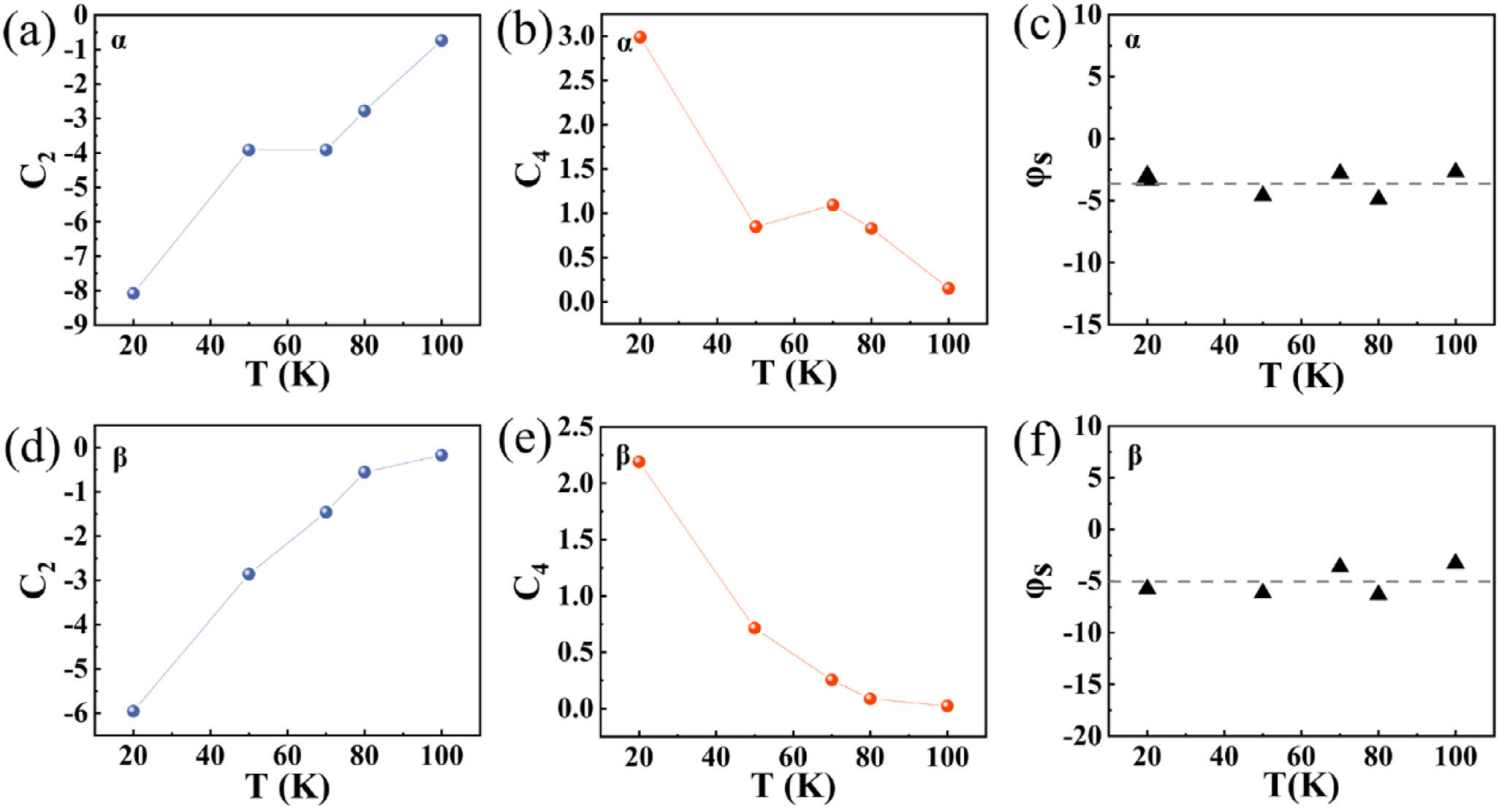
The fitting parameters obtained based on the AMR results at different temperatures. a–c) show the fitted AMR data for the *α*‐plane. d–f) present the fitted parameters for the *β*‐plane.

For simplicity, the AMR signal is fitted using the expression:

(10) $$\begin{array}{ccc} & & C_{2}cos\left(2\left(\theta_{AMR}+\varphi_{S}\right)\times \frac{3.14}{180}\right)\\ & & \;+\;mC_{4}cos\left(4\times \left(\theta_{AMR}+\varphi_{S}\right)\times \frac{3.14}{180}\right)+A \end{array}$$

where θ_*AMR*_ is the rotation angle and φ_*S*_ is a phase offset angle relative to the symmetry axes nominally at 90° and 270°. As temperature increases, *C*_2_ increases steadily (**Figure** **6**a,d), while the fourfold coefficient *C*_4_ decreases (Figure 6b,e). The twofold symmetry dominates the AMR signal, and more so at higher temperatures. The microscopic origin of such fourfold symmetric AMR remains an active research topic.^[^ ^52^, ^53^, ^54^ ^]^ Considering that Mn_3_Si_2_Te_6_ crystallizes in a structure analogous to Bismuth and MnBi_2_Te_4_^[^ ^53^ ^]^ and exhibits half‐metallicity,^[^ ^25^ ^]^ the observed unusual fourfold AMR most likely originates from the topological orbital moment of magnetic Bloch electrons,^[^ ^53^ ^]^ rather than from relaxation time anisotropy^[^ ^52^ ^]^ or high‐order term of spin‐orbit interactions^[^ ^54^ ^]^ in a cubic system. Furthermore, the phase offset parameter φ_*S*_ reveals peak shift of ≈5° in the *β*‐plane and 3.6° in the *α*‐plane, respectively (Figure 6c,f), suggesting that the MR maxima occur when the strong magnetic field is rotated ≈ 3° to 5° beyond the principle symmetry axes (90° and 180°). These shifts provide further evidence that the magnetic moments in the Mn_3_Si_2_Te_6_ are tilted with respect to the *ab*‐plane.

## 3 Conclusion

High‐quality Mn_3_Si_2_Te_6_ single crystals were synthesized via the solvent‐flux method. Magnetic characterization revealed that the magnetization along the easy axis within the *ab*‐plane did not fully saturate, and that the material exhibits anomalous MR in the ferrimagnetic state. Specifically, when a magnetic field was applied along the hard magnetization *c*‐axis, Mn_3_Si_2_Te_6_ exhibited behaves consistent with a conventional 3D ferrimagnet. Once the demagnetizing field was overcome, the alignment of spin polarization with the magnetic moments suppressed spin‐disorder scattering, resulting in a large negative saturation MR. Conversely, for in‐plane magnetic fields, the MR remained unsaturated, attributed to persistent spin misalignment caused by a *c*‐axis component of the magnetization. AMR measurements further revealed distinct angular evolution patterns under in‐plane versus out‐of‐plane fields, consistent with a quasi‐2D transport character and tilted spin structure. Our findings not only deviate from traditional frameworks based on double exchange or Jahn‐Teller distortions mechanisms, but also challenge the applicability of the COC model in Mn_3_Si_2_Te_6_ single crystals system. The results, establish a new framework for manipulating magnetotransport in layered magnets. This study advances the fundamental understanding of the anomalous anisotropic CMR effect in Mn_3_Si_2_Te_6_ and highlights its potential for future spintronic applications.

## 4 Experimental Section

High‐purity single crystals were synthesized employing an improved self‐flux growth methodology. Stoichiometric amounts of high‐purity Mn (99.99%), Si (99.999%), and Te (99.999%) were precisely weighed in a molar ratio of 3:2:12 and sealed in an alumina crucible. The crucible was then sealed within a quartz tube under a high vacuum of 10^−5^ Pa. The sample was heated to 1000 °C at a rate of 100 °C h^−1^, held for 24 h to ensure complete melting and homogenization in a muffle furnace. Subsequently, the sample was cooled at 2 °C h^−1^ from 1000 to 700 °C over 150 h to promote crystal nucleation and directional growth. The crucible was quickly removed from the furnace, and the flux was separated from the crystals by centrifugation, yielding well‐defined hexagonal plate‐like single crystals up to centimeter‐sized as shown in the inset of Figure 1a.

The sample structure was characterized using powder X‐ray diffraction (XRD, Rigaku SmartLab) equipped with Cu‐Kα radiation. Elemental distribution analysis was conducted via scanning electron microscopy (SEM, JEOL JSM‐7500F) coupled with energy‐dispersive X‐ray spectroscopy (EDS). To investigate the magnetic and electrical transport properties of Mn_3_Si_2_Te_6_, comprehensive characterization was carried out using a physical property measurement system (PPMS DynaCool, 9T, Quantum Design). The MR measurements were performed employing the standard four‐point method. Magnetization measurements were performed using a vibrating sample magnetometer in a Physical Property Measurement System (PPMS‐14T, Quantum Design) with fields applied both parallel to the *ab*‐plane and along the *c*‐axis. High‐magnetic‐field measurements were conducted at the Wuhan National High Magnetic Field Center. The Zero‐Field Cooled (ZFC) and Field Cooled (FC) magnetization curves of a Mn_3_Si_2_Te_6_ single crystal were measured over a temperature range of 2–380 K. For the ZFC protocol, the sample was first cooled from 380 to 2 K in the absence of an external magnetic field. A probe field of 100 Oe was then applied, and the magnetization was recorded while warming the sample to 380 K. For the FC curve, the sample was cooled from 380 to 2 K under a static magnetic field of 1000 Oe, and the magnetization was subsequently measured during the warming cycle from 2 to 380 K under the same field.

## Conflict of Interest

The authors declare no conflict of interest.

## Supporting information



Supporting Information

## Data Availability

The data that support the findings of this study are available from the corresponding author upon reasonable request.
