# Supplementary material for: Mechanism of Anomalous Anisotropic Colossal Magnetoresistance in Quasi‐2D Mn3Si2Te6 Bulk Single Crystal
Source: Adv Sci (Weinh). 2025 Oct 14;13(2):e14651. doi: 10.1002/advs.202514651 (PMC12786375; doi:10.1002/advs.202514651)
Supplement: Supplementary file 1 — Supporting Information [file ADVS-13-e14651-s001.docx]

Supporting Information

Mechanism of Anomalous Anisotropic Colossal Magnetoresistance in Quasi-2D Mn_3_Si_2_Te_6_ Bulk Single Crystal

*Shiqi Li, Xiong He, Shuai Li, Tianyi Li, Wenhao Zhang, Lizhi Yi, Guangduo Lu, Zhengcai Xia, Yunli Xu*,* *John Q Xiao*, Liqing Pan**

**1. The local crystal structure diagram of the Mn_3_Si_2_Te_6_ single crystal.**


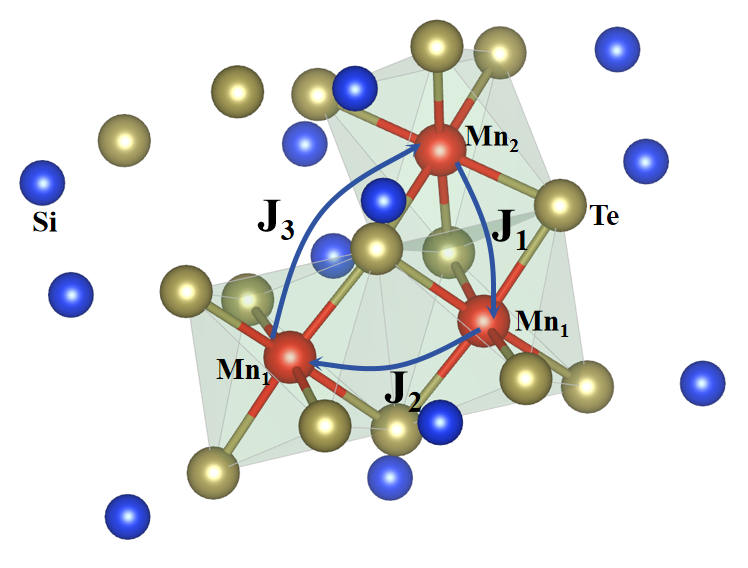


Figure S1 Local crystal structure diagram of the Mn_3_Si_2_Te_6_ single crystal.

**2. Curie-Weiss analysis results.**


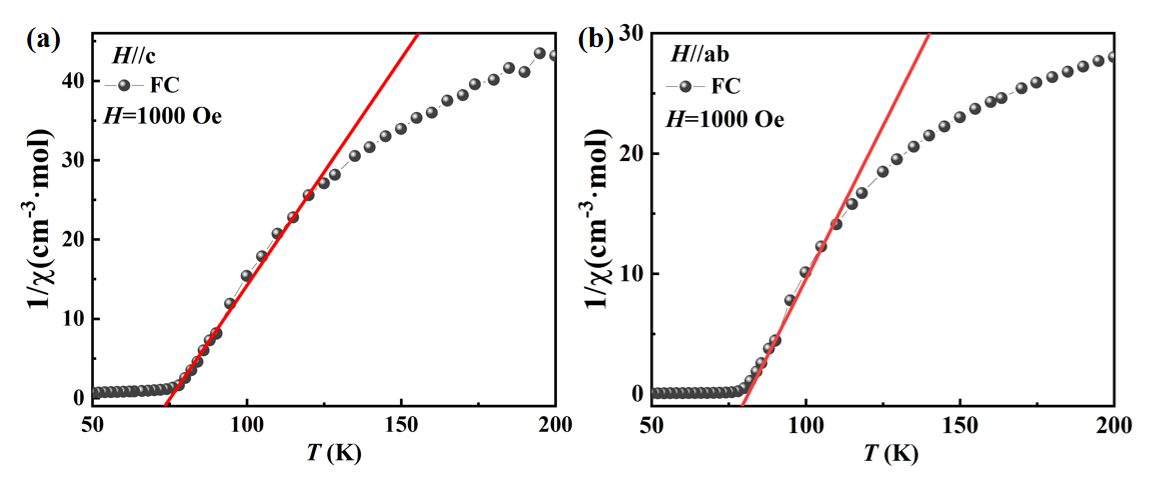


Figure S2 The FC curve is fitted using the Curie-Weiss law in the a) *H*//c-axis, and b) *H*//*ab*-plane, respectively.
